# Oxaliplatin-Induced Thrombocytopenia: A Case Report and Review of Pathophysiology of Various Speculative Mechanisms

**DOI:** 10.7759/cureus.9929

**Published:** 2020-08-21

**Authors:** Haider Ghazanfar, Iqra Nawaz, Nisha Ali

**Affiliations:** 1 Internal Medicine, BronxCare Health System, Bronx, USA

**Keywords:** adenocarcinoma, chemotherapy, oxaliplatin, thrombocytopenia, morbidity, mortality

## Abstract

Oxaliplatin is one of the most common anti-neoplastic agents used in the treatment of small bowel adenocarcinoma. Peripheral neuropathy is one of the most common side effects of oxaliplatin. Oxaliplatin-induced thrombocytopenia is an extremely rare side effect and can result from various mechanisms. We present a case of a 66-year-old female who presented to the hospital for the ninth cycle of FOLFOX chemotherapy for her small bowel adenocarcinoma. The patient developed severe thrombocytopenia within 24 hours of administration of oxaliplatin. Physicians need to be aware of the sudden onset of severe thrombocytopenia associated with oxaliplatin use as early diagnosis and prompt treatment can prove lifesaving for these patients.

## Introduction

Oxaliplatin is a third-generation, platinum-based anti-neoplastic agent that was first discovered in 1976. It is considered amongst the safest and the most effective medicine by the World Health Organization (WHO). It is used to treat colorectal and small bowel cancer in conjunction with 5-fluorouracil (5-FU) and folinic acid; a combination named FOLFOX [1].

Peripheral neuropathy is one of the most common side effects of oxaliplatin. The incidence of grade 3 and 4 neuropathy is about 15% [2]. Common gastrointestinal manifestations of oxaliplatin include nausea, vomiting, and diarrhea. Hypersensitivity reactions have also been reported in 10%-15% of patients after receiving multiple cycles of the FOLFOX regimen [3]. Patients taking oxaliplatin can develop moderate to severe neutropenia and about 4% of patients will experience neutropenic fever [2, 4]. Oxaliplatin-induced thrombocytopenia is a rare complication and only 3%-4% of the patient will develop grade 3 and 4 thrombocytopenia [5]. We present a case of a 66-year-old female who developed oxaliplatin-induced thrombocytopenia.

## Case presentation

**Figure 1:**
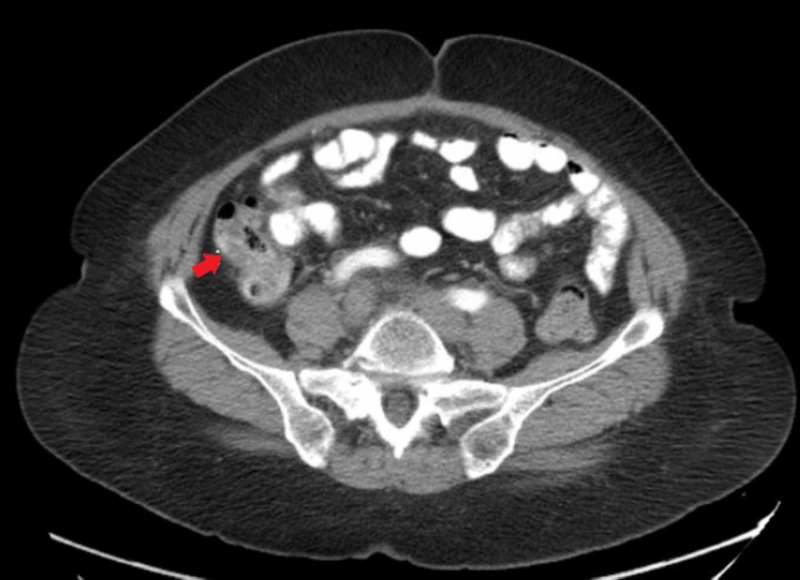
CT abdomen and pelvis with contrast.

The patient is a 66-year-old female who presented to the hospital for the ninth cycle of FOLFOX chemotherapy. FOLFOX is a chemotherapy regimen which comprises folinic acid (leucovorin), 5-FU, and oxaliplatin. The patient's past medical history was significant for small bowel (jejunal) adenocarcinoma with mesenteric metastasis diagnosed in December 2017. At the time of diagnosis, the patient was found to have invasive moderately differentiated stage IV [T3NOM1] adenocarcinoma. There is no standard first-line chemotherapy approach for small bowel cancers and patients are usually treated on colon cancer treatment paradigms. The patient was given her first cycle of chemotherapy with FOLFOX in February 2018. The patient was treated with 12 cycles of FOLFOX chemotherapy from February 2018 to July 2018. In July 2018 oxaliplatin dose was reduced for peripheral neuropathy. As there was no clear radiologic manifestation of residual disease at that time the therapy was switched to fluorouracil/leucovorin (Roswell Park Schema) with the plan of re-introduction of FOLFOX if the disease progresses. The patient underwent CT of the chest, abdomen, and pelvis at regular intervals. The patient underwent CT of abdomen and pelvis in June 2019 which revealed a complex solid and cystic lesion/mass in the right pelvis, increase in the size of scattered peritoneal implants, and mild mesenteric adenopathy. This has been shown in Figure 1.

The patient was restarted on FOLFOX in June 2019. The patient’s platelet counts at that time were 256 k/uL. The patient's clinical course since then had been complicated by chemotherapy-induced nausea and vomiting, iron deficiency anemia, peripheral neuropathy, and alopecia. The patient had no known drug allergies. The patient denied smoking, drinking alcohol, or using illicit substances.

The patient was admitted to the oncology floor for chemotherapy. The patient’s platelets at the time of admission were 240 k/uL. The patient’s comprehensive metabolic profile was normal. On the second day of admission, the patient developed gross hematuria with the passage of the clot. There was no other source of active bleeding. The patient’s platelet counts were repeated and were found to be 5 k/uL. Her hemoglobin and hematocrit remained stable at 13 g/dL and 38% respectively.

**Figure 2:**
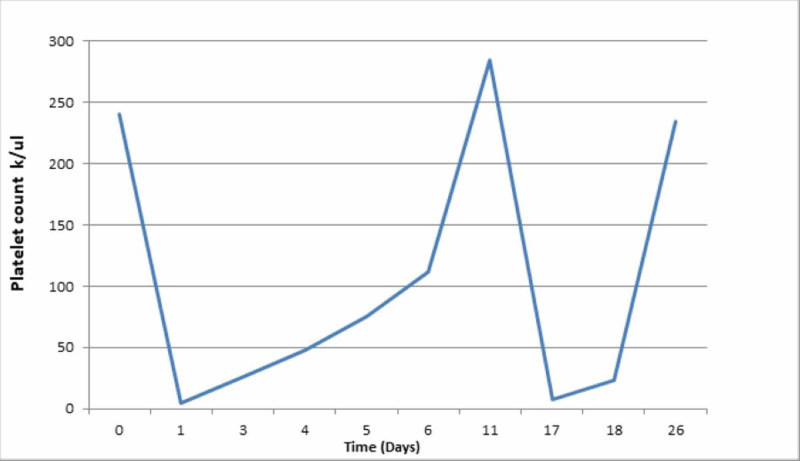
Trend of platelet count during the hospital course.

The critical care team was consulted and the patient was accepted to the critical care unit for further management. Fluid resuscitation was done. The patient was transfused six units of platelets. Chemotherapy was discontinued and heparin was switched to argatroban. Heparin-induced thrombocytopenia (HIT) panel was sent and was found to be negative. The patient had no further episodes of active bleeding or gross hematuria. The patient’s platelet count slowly improved and returned to the baseline at 285 k/uL on the 11th day of admission. This has been shown in Figure 2.

The patient was transferred to the medicine floor for further management. After the resolution of thrombocytopenia, oxaliplatin dose was reduced to 105 mg/mL and the dose was administered six days after persistent normalization of platelet count. However, within 24 hours of dose administration, we noticed immediate recurrent severe thrombocytopenia with subsequent development of vaginal bleeding. The platelet count was dropped from 285 to 8 k/uL. The patient received six units’ platelets and fluid resuscitation was done with clinical improvement. Hemoglobin/Hematocrit remained stable without any significant drop. Disseminated intravascular coagulation and hemolytic workups were normal. The leukocytic count remained normal. At this point, acute onset of oxaliplatin-induced severe immune thrombocytopenia was suspected and oxaliplatin was discontinued completely. The patient’s chemotherapy regiment was changed to FOLFRI.

## Discussion

Oxaliplatin-induced thrombocytopenia is a rare complication. Multiple theories have been described to understand the precise mechanism of immuno-hematologic manifestations of this neoplastic agent. These mechanisms are summarized in Table 1.

**Table 1:** Mechanisms causing oxaliplatin-induced thrombocytopenia.

Bone marrow suppression
Splenic sequestration
Thrombocytic thrombocytopenic purpura
Hemolytic uremic syndrome
Disseminated intravascular hemolysis
Immune thrombocytopenic purpura
Hypersensitivity reactions

Bone marrow suppression is a well-known complication related to chemotherapy. Oxaliplatin-induced thrombocytopenia due to bone marrow suppression develops in about 45%-77% of colorectal cancer patients who receive oxaliplatin [6]. Bone marrow suppression can be suspected if the patient develops concomitant thrombocytopenia and neutropenia. Patients who develop thrombocytopenia through this pathophysiologic mechanism usually have a sub-acute onset and present one week following chemotherapy. Bleeding and hypersensitivity are not commonly seen in these patients. The gold standard test of diagnosis is bone marrow aspirates which would show toxicity to megakaryocytic progenitors [6].

The second theory related to oxaliplatin-induced thrombocytopenia suggests that oxaliplatin might have some hepatotoxic effects resulting in hepatic sinusoidal injury and fibrosis leading to sinusoidal obstruction, veno-occlusive lesions, and nodule regenerative hyperplasia. These findings have been seen in autopsy findings of patients treated with oxaliplatin [3]. According to a study patients receiving oxaliplatin developed noncirrhotic portal hypertension and it was mostly attributed to oxaliplatin-induced hepatic injury [7]. The resultant portal hypertension leads to the development of esophageal varices, ascites, and splenomegaly which eventually becomes the mode of sequestration of platelets. This pathophysiological mechanism of oxaliplatin-induced thrombocytopenia usually has a subacute onset and these patients usually do not develop life-threatening bleeding. Imaging evidence of portal hypertension and splenomegaly is the mainstay for the diagnosis [8].

Thrombotic thrombocytopenic purpura (TTP) and hemolytic-uremic syndrome (HUS) are rare complications associated with oxaliplatin. Acute onset back pain, dark urine, and oliguria were the most common clinical presentation among these patients [9]. According to a systemic review, about 52% of patients with oxaliplatin immune-induced syndrome had associated grade 4 thrombocytopenia, and while 6.6% of the patients had grade 4 anemia [10]. In the same study, about 87% of the patients with oxaliplatin immune-induced syndrome were found to have either positive Coombs test or anti-platelet antibody; suggesting oxaliplatin-induced immune system activation [10]. Oxaliplatin-induced disseminated intravascular hemolysis resulting in life-threatening thrombocytopenia has also been reported, the most common finding among these patients was positive Coombs test [11].

Oxaliplatin-induced immune thrombocytopenia (OITP) is another proposed theory and an increasing number of OITP cases are reported. OITP is mostly a clinical diagnosis. This immune-mediated cascade results in the formation of drug-dependent IgG antibodies against platelets membrane glycoproteins causing the destruction of platelets [12-13]. Patients who develop thrombocytopenia through this mechanism usually present with acute onset isolated drop in platelet count right after the administration of oxaliplatin. These patients usually develop life-threatening bleeding. Flow cytometry and enzyme-linked immunosorbent assay (ELISA) are two tests that can be used for diagnostic purposes. Flow cytometry technique can identify the presence of oxaliplatin-induced antibodies and ELISA monoclonal antibody test determines the platelet glycoproteins; such as GPIIb/IIIa or GPIb/IX/V [12]. It is very crucial to differentiate OITP from other causes of isolated thrombocytopenia which include autoimmune thrombocytopenia, post-transfusion purpura, and platelet transfusion refractoriness. The temporal association of drug exposure and thrombocytopenia can help in establishing the diagnosis. Immediate withdrawal of the drug is the mainstay of treatment. In some cases, increased levels of inflammatory markers such as TNF alpha IL10, and IL 6 have also been reported [14]. The administration of steroids and antihistamines has been used to reduce the levels of these inflammatory cytokines [15-16]. In our patient, we noticed a sudden onset of isolated thrombocytopenia which resolved upon discontinuation of oxaliplatin. Oxaliplatin-directed antibodies were not tested due to unavailability. We suspected OITP as a cause of thrombocytopenia in our patient because of the clinical presentation and course.

 Oxaliplatin-induced cumulative dose effect causing peripheral neuropathy is very well documented. Cumulative dose effects result in chronic neuropathy as opposed to acute neuropathy and are most commonly seen in patients who have received a total dosage of more than or equal to 540 mg/m2 [17]. Oxaliplatin is renally excreted and its clearance rate is directly proportional to glomerular filtration rate (GFR). Impaired GFR is associated with reduced plasma clearance of oxaliplatin. The second major mechanism of platinum elimination from systemic circulation is tissue distribution [18]. This tissue distribution of platinum metabolites may explain the mechanism of oxaliplatin cumulative dose effects. Dose reduction/modification strategies have been implicated to reduce oxaliplatin-induced neuropathy. The “stop and go” treatment strategy has been associated with a lower rate of neurotoxicity [19]. In this strategy, oxaliplatin is given for a shorter period of time and is reintroduced into the chemotherapy regimen after an oxaliplatin-free interval.

Oxaliplatin-induced immune thrombocytopenia could also be another manifestation of a cumulative dose effect. However, a dose reduction strategy might not be helpful in this case. In our patient after the first occurrence of thrombocytopenia, the oxaliplatin dose was reduced from 105 to 65 mg/mL. Despite the reduction in the dose our patient still developed severe thrombocytopenia associated with massive bleeding leading to a complete discontinuation of oxaliplatin therapy in our patient. 

## Conclusions

In conclusion, sudden onset of severe thrombocytopenia resulting in life-threatening bleeding is a rare yet very serious complication associated with oxaliplatin. The exact mechanism of oxaliplatin-induced thrombocytopenia is obscure although multiple theories have been suggested. There is a need to do further researches at the molecular level for a better understanding of pharma-kinetics and the immune-inflammatory mechanism of oxaliplatin-induced thrombocytopenia. Oxaliplatin desensitization methods need to be further evaluated to reduce the effects of OITP. It is of paramount importance that treating physicians should have a high suspicion of such oxaliplatin-induced side effects and understanding of its mechanism for prompt diagnosis and treatment, as it is essential in avoiding its life-threatening complications.
